# Prevalence and Predictors of Contraceptives Use among Women Aged (15–49 years) with Induced Abortion History in Ghana

**DOI:** 10.1155/2020/2630905

**Published:** 2020-08-28

**Authors:** Mohammed Gazali Salifu, Kamaldeen Mohammed

**Affiliations:** ^1^Ministry of Health, Ghana, Post Office Box M44, Accra, Ghana; ^2^Department of Geography, Western University, 1151 Richmond Street, N6A 3K7, London, Ontario, Canada

## Abstract

**Background:**

The incidence of abortion in Ghana ranges from 27 per 1000 to 61 per 1000 women, causing gynecological complications and maternal mortality. The use of modern contraceptives and its associated factors among women aged 15–49 years have been documented. However, utilization of modern contraceptives specifically among women with induced abortion history is underreported. This study therefore aimed at determining the proportion and identifying predictors of contraceptives use in this underreported population.

**Methods:**

This study used secondary data from the 2017 Ghana Maternal Health Survey (GMHS) for the analysis. The analysis is on a weighted sample of 3,039 women aged (15–49 years) with a history of induced abortion. Both descriptive and inferential methods were employed. The chi-square test, univariate and multivariate logistic regression techniques were used to assess statistical associations between the outcome variable and the predictors. Statistical significance was set at 95% confidence interval and *p* values ≤0.05.

**Results:**

Out of the 3,039 participants, 37% (95% CI: 34.6, 38.84) used contraceptives. We identified women' age, union, place of residence, knowledge of fertile period, total pregnancy outcomes, and region as strong significant (95% CI, *p* ≤ 0.05) predictors of post induced abortion contraceptives use.

**Conclusion:**

Contraceptives use among this vulnerable population is low. Therefore, there is a need to provide widespread access to postabortion contraception services and enhance efforts to efficiently integrate safe abortion practices law into health services in Ghana.

## 1. Introduction

Globally, the proportion of unsafe abortions in developing countries is 49.5% [1]. Sub-Saharan Africa (SSA) accounts for 61% of all deaths due to unsafe abortion with a case fatality rate for Africa in 2008 approximated at 520 per 100,000 unsafe abortions [2]. In Ghana, 20% of women in reproductive age (15–49 years) have experienced induced abortion during their lifetime (ICF, Ghana Health Service (GHS), 2018). Incidences of abortion in Ghana range from 27 per 1000 to 61 per 1000 women [3]. Admissions to gynecological wards and maternal mortality can be attributed to induced abortions in Ghana [4].

Despite the 1985 Ghanaian law accommodating safe abortion practice on certain medicosocial grounds, there have been observed delays in the formulation and implementation of policies to integrate the law into health services [5]. Factors such as lack or insufficient knowledge on safe abortion, stigma related to unintended pregnancy, religious beliefs, and implicit nature of the abortion law have significantly influenced safe abortion practice negatively in Ghana [5, 6].

These challenges associated with the delays in the implementation of comprehensive abortion care in Ghana make the use of modern contraceptives imperative and a reliable option towards reducing the high rates of unwanted pregnancies [7–9], induced abortions [9], maternal deaths [10], and sexually transmitted infections (STIs) [8].

The use of modern contraceptives and its associated factors among women age (15–49 years) have been documented in Ghana [9, 11–16]. However, utilization of modern contraceptives specifically among women with induced abortion history is underreported despite their vulnerability. Relatedly, only 19% of women aged 15–49 years with a history of induced abortion receive contraception support in Ghana (ICF, Ghana Health Service (GHS), 2018). In addition, several studies reported in Ghana and elsewhere have highlighted varying proportions and predictors of contraceptives use among women with induced abortion history [17–20].

This study, therefore, used a nationally representative survey [21] to report the national proportion and identify factors significantly influencing the use of modern contraceptives among this age group of women (15–49 years) with induced abortion history. This will provide valuable information for policymakers and implementers to make informed and evidence-based decisions regarding policy formulation and implementation on reproductive health policies and programs.

## 2. Materials and Methods

### 2.1. Study Design and Data Source

This study is a retrospective cross-sectional design using the Ghana Maternal Health Survey (GMHS) conducted in 2017. The GMHS is a nationally representative special survey, which collects data relating to women, children, birth, household, and other feminine variables covering all regions and districts of the country. The survey was first conducted in 2007 and also in 2017.

### 2.2. Sampling Approach and Study Population

For this current Ghana Maternal Health Survey (GMHS) 2017, 27,001 households were selected for the sample, of which 26,500 were occupied at the time of fieldwork. Data were collected from participants with a questionnaire through a multistage probability sampling technique. During the first stage, proportional sampling was used to select 900 enumeration areas (EAs) (including 466 EAs in urban and 434 EAs in rural areas, respectfully) to reflect the sizes of EAs with independent sampling in each stratum. Secondly, a household listing was carried out from 25 January to 9 April 2017 in all the selected EAs to serve as a sampling frame for the selection of households in the second stage. The interviewed households were 26,324, resulting in a response rate of 99%. With the interviewed households, 25,304 eligible women were recruited for individual interviews. However, interviews were completed with 25,062 women. The response rate of the completed interview was 99%. The sampling procedure is extensively detailed in the complete final report of the Ghana Maternal Health Survey 2017 (ICF, Ghana Health Service (GHS), 2018) [21]. In this present study, our analysis is on a weighted sample of 3,039 women aged 15–49 years out of the 25,062 reproductive age women data analyzed in the 2017 GMHS.

### 2.3. Inclusion Criteria

The study included women in their reproductive ages between 15 and 49 years who have ever been in a situation in which she or someone else had to do something to end her pregnancy.

### 2.4. Exclusion Criteria

The exclusion criteria were those who met the inclusion criteria above but had missing data.

### 2.5. Outcome Variable

The outcome variable was “current usage of any method of contraceptives (modern or traditional) to delay or avoid getting pregnant.” The responses were coded as either “Yes” or “No.”

### 2.6. Independent Variables Measures

We included several theoretical pertinent sociodemographic variables. Women age group (14–19, 20–24, 25–29, 30–34,35–39, 40–44, and 45–49), religious status (Christianity, Islam, traditional, and others), age at first intercourse (<16 and 16 or above), health insurance (yes or no), knowledge of fertility (yes or no), current union (yes or no), region (Eastern, Greater Accra, Northern, Upper East, Upper West, Volta, Eastern, Central, Ashanti, Western, and Brong Ahafo), education (primary, JHS, SHS or above, and no education), place of residence (rural or urban), and gravidae (1–3, 4-5, and 6 or more). Furthermore, variables were recoded where appropriate to produce a meaningful sample for the analysis.

### 2.7. Statistical Analysis

This analysis used both descriptive and inferential methods. Descriptive statistics used are mainly frequencies and percentages. Chi-square tests, univariate and multivariable techniques were used to assess statistical associations between the outcome variable and the predictors.

The chi-square test of independence was used to statistically identify factors associated with the outcome variable looking at their estimated confidence intervals (CI), and *p* values less than or equal to 0.05 were used to retain and include the variables in a univariate logistic regression analysis. In the univariate analysis, variables with *p* values <0.05 were simultaneously included in a multivariable logistic regression model to generate adjusted odds ratio (AOR) with their corresponding 95% confidence intervals.

This statistical approach was implemented in STATA (Stata Statistical Software: Release 16. College Station, TX: StataCorp LP) software. The complex survey command “svy” was used to estimate means, proportions, and confidence intervals (CI). The final model goodness of fits was checked using the “svylogitgof” command [22]. The results revealed no evidence of a lack of fit with our model in significantly predicting contraceptives use.

### 2.8. Ethics

The Ghana Health Service Institutional Review Board (IRB) approves the Demographic Health Surveys study protocol, survey instruments, and materials before commencement of the surveys. Individual consent was also obtained during the data collection process. Data were then used after approval was obtained. Furthermore, this study concept was presented to ICF for permission to use the datasets in this study, and approval was duly granted before its use. All terms of use have been observed.

## 3. Results

### 3.1. Descriptive Statistics

A total of 3,039 women met the inclusion criteria for the study. The mean and standard deviation age in years of the women was 32.93 (8.32). Almost 37% (95% CI: 34.6, 38.84) of the participants used contraceptives. The mean and standard deviation age in years of respondents using contraceptives was 33.11 (8.09). The participants who had education above primary school level were 73.43%. Approximately, 67% of participants were in union (either married or cohabitating with a man). Majority of participants were Christians (91.16%), and 68.35% of participants lived in urban areas. Greater Accra and Ashanti regions cumulatively constituted 51.1% of all participants (Table 1).

In Table 2, 770 (70.33%) of contraceptives users were in a union, and 393 (37.71%) and 737 (62.29%) of participants using contraceptives lived in rural and urban areas. Among participants using contraceptives, 1,031 (91.87%) were Christians. 347 (31.86%) contraceptives users had sexual intercourse below age 16. Furthermore, 941 (83.19%) of contraceptives users were registered with health insurance (Table 2).

### 3.2. Predictors of Contraceptives Use

Chi-square tests revealed respondents' current age (95% CI: *χ*^2^ = 106.90, *p* ≤ 0.001), union (95% CI: *χ*^2^ = 11.56, *p* ≤ 0.05), educational level (95% CI: *χ*^2^ = 17.6039, *p* ≤ 0.01), residence (95% CI: *χ*^2^ = 29.89, *p* ≤ 0.001), knowledge of the fertile period (95% CI: *χ*^2^ = 14.74, *p* ≤ 0.001), and gravidae (95% CI: *χ*^2^ = 18.88, *p* ≤ 0.001) to be significantly associated with contraceptives use (Table 3).

A univariate and multivariate logistic regression was used to identify further significant predictors of contraceptives use (Table 3). At the univariate level, women aged 30–34 (OR = 0.61, 95% CI: 0.38–0.98, *p* ≤ 0.05), 35–39 (OR = 0.51, 95% CI: 0.31–0.83, *p* ≤ 0.01), 40–44 (OR = 0.37, 95% CI: 0.22–0.62, *p* ≤ 0.001), and 45–49 (OR = 0.30, 95% CI: 0.18–0.48 were significantly less likely to use contraceptives compared with women aged 15–19 (Table 3). Participants in a union were 1.32 (CI: 1.09–1.59, *p* ≤ 0.005) times significantly more likely to use contraceptives than those not in a union. Also, participants from Ashanti (OR = 1.82, 95% CI: 1.21–2.74, *p* ≤ 0.005), Northern (OR = 2.20, 95% CI: 1.19–4.08, *p* ≤ 0.05), Brong Ahafo (OR = 2.06, 95% CI: 1.28–3.31, *p* ≤ 0.005), and Eastern (OR = 2.27, 95% CI: 1.47–3.49, *p* ≤ 0.001) regions were more likely users of contraceptives compared to participants from Central region (Table 3).

Also, the probability of using contraceptives among participants with SHS level of education was 0.74 (95% CI: 0.55–0.99, *p* ≤ 0.05) times less likely compared to those with only primary level of education. Rural dwellers were 1.55 (95% CI: 1.26–1.90, *p* ≤ 0.001) times more likely users of contraceptives compared with urban dwellers. Women who had their total pregnancy outcomes between 4 and 5 were 0.68 (95% CI: 0.55–0.84, *p* ≤ 0.001) times less likely to use contraceptives compared with women with 1–3 total pregnancy outcomes (gravidae) (Table 3). Furthermore, women who knew their fertile period were 1.63 (95% CI: 0.38–0.98, *p* ≤ 0.05) times more likely to use contraceptives than those who do not know. At the multivariate level, age of respondents, union, region, residence, gravidae, and knowledge of the fertile period remained significant predictors while educational level became insignificant predictor after controlling for other predictive factors (Table 3).

## 4. Discussions

This study was carried out to estimate the proportion and identify predictors of contraceptives use among women with induced abortion history. We estimated 36.69% (95% CI: 34.6, 38.84) contraceptives use in this population. The estimated proportion is lower compared to similar studies carried out by Makenzius et al., Mekuria et al., and Opoku [18–20]. However, a study carried out in Western Nigeria showed a much lower proportion of contraceptives use [17]. The differences in proportions observed may be attributed to the differences in medical counselling and guidance services received on contraception after abortion episodes, as women tend to take up contraception when counselled properly after any abortion episode [23].

The findings also revealed that older women aged 30–34, 35–39, 40–44, and 45–49 are less likely to use contraceptives relative to younger ones (15–19 years); this corroborates a study conducted by Abebe [24]. However, our finding is contrary to studies carried out by Mekuria et al. and Maxwell et al. [19, 25]. Adolescents have been identified as a highly sexually active group; so, many health advocacy programs in Ghana, especially those on contraception have focused on them, so this might be responsible for the observed contraceptives use differences identified across the various age groups. Women aged 20–29 years have also been reported to have the highest proportion of abortions in most countries [26]. There is the need for strategies to increase contraception uptake through counselling among older women, improve access to postabortion services, and enhance health service provider training and supervision on postabortion contraception [18]. Also, stigma by health service providers affects women contraception uptake after induced abortion and needs to addressed as well [27].

This study finding also observed that participants in union (married or cohabitating) have higher odds of using contraceptives than those not in a union, which is similar to other studies [18, 28–31]. Other studies have also observed contraceptives use among married women when they have at least eight deliveries and birth intervals less than 24 months between two children [32]. In addition, male involvement in family planning consultation increases their responsibility for contraception [32].

Women living in rural areas had higher odds of contraception than those in urban areas. However, rural women access to maternal and child health services are lowest compared to those in urban areas [33]. In addition, effective postabortion care services are mostly inadequate in villages [34]. Nonetheless, urban women contraception use is low, and a significant proportion of them also experience reproductive ill-health [35], but generally, contraception uptake among rural women in Ghana relative to urban women is on the increase [12, 15]. Furthermore, our study revealed that women who know their fertile period are more likely to use contraceptives than those who do not know. This may be attributed to the higher conception odds during this window; however, a study conducted in Nigeria among adolescents has pointed out that not all women restrict intercourse to the safe periods of their cycle [36]. Moreover, there might be other factors that influence unsafe sex practice during this period, which can be exploited further.

While acknowledging the importance of all these predictors highlighted, there is the need for broader methods to satisfy clients varieties and differences regarding contraception [37], effectively addressing the effects of social norms on postabortion contraception, enhanced postabortion counselling services, and encouraging multisectoral collaborations in postabortion-related contraception programs formulation and implementation.

### 4.1. Strengths of the Study

This study is from a nationally representative sample and makes room for generalizability of study findings across Ghana. Also, Demographic and Health Surveys (DHS) are planned properly and well-executed; hence, the data are of high quality. Furthermore, observations with complete dataset meeting the study criteria was large.

### 4.2. Limitations of This Study

Firstly, the data used for this study were obtained through a cross-sectional study design, hence preventing causations from being inferred. The survey [21] obtained information retrospectively, which has a recall bias since participants self-reported, which spanned a 5-year period prior to the survey. Recall bias has an immense effect on coefficient estimates and overall significant testing, and so, interpretations/use of the findings should be performed cautiously. Finally, we did not control for all potential confounders in our analysis.

## 5. Conclusion

We explored the proportion of contraceptives use and its associated significant predictors among women aged 15–49 years with a history of induced abortion. The proportion was low at 37%. Women age, union, place of residence, knowledge of fertile period, gravidae, and region were identified as significant predictors of contraceptives use. We recommend that the Ministry of Health and Ghana Health Service facilitate widespread and easy access to modern contraceptives, provide postabortion contraception services, including guidance and counselling to women who need these services. In addition, we recommend efforts to be enhanced in the formulation and implementation of policies by the Government of Ghana to effectively and efficiently integrate the abortion law into services, and lastly, we recommend that the National Health Insurance Scheme (NHIS) should absorb contraception services as a way of curbing high cost as a potential predictor of its low uptake

## Figures and Tables

**Table 1:** Background characteristics of respondents (*N* = 3,039).

Variable	Frequency	Percentage
Age		
15–19	115	3.516
20–24	446	14.45
25–29	629	20.85
30–34	576	18.74
35–39	515	17.36
40–44	399	13.54
45–49	359	11.53

Educational level		
Primary	503	16.68
JHS	1222	41.29
SHS and above	1011	32.14
No education	303	9.891

Religious distribution		
Christianity	2751	92.16
Islam	244	6.276
Traditional	8	0.2365
Others	36	1.325

Contraceptives use		
Yes	1130	36.69
No	1909	63.31

Residence		
Rural	925	31.65
Urban	2114	68.35

Union		
Yes	2002	66.51
No	1037	33.49

Region		
Western	388	13
Central	248	8.125
Greater Accra	567	26.05
Volta	212	6.005
Eastern	364	10.62
Ashanti	644	25.05
Brong Ahafo	383	9.493
Northern	86	0.7622
Upper East	69	0.4839
Upper West	78	0.4042

**Table 2:** Distribution and chi-square analysis of outcome (contraceptives use) across respondents' characteristics (sociodemographic, socioeconomic, and abortion-related characteristics).

Variable	Contraceptives use	
	No (%)	Yes (%)
Age		
*X*2 = 106.8983, *p* ≤ 0.001		
15–19	61 (2.84)	54 (4.674)
20–24	224 (11.66)	222 (19.28)
25–29	347 (18.31)	282 (25.23)
30–34	363 (18.75)	18.73 (18.73)
35–39	334 (18.42)	181 (15.54)
40–44	298 (15.84)	101 (9.59)
45–49	284 (14.18)	77 (6.96)

Union		
*X*2 = 11.5593, *p* ≤ 0.005		
Yes	1232 (64.29)	770 (70.33)
No	677 (35.71)	360 (29.67)

Educational level		
*X* = 17.6039, *p* ≤ 0.05		
Primary	320 (16.22)	183 (17.45)
JHS	727 (39.73)	495 (44)
SHS	683 (34.81)	328 (27.54)
No education	179 (9.24)	124 (11.01)

Religion		
*X* = 0.8010, *p* > 0.05		
Christianity	1720 (92.33)	1031 (91.87)
Islam	162 (6.23)	82 (6.35)
Traditional	4 (1.87)	4 (0.32)
Others	23 (1.25)	13 (1.45)

Residence		
*X* = 29.8850, *p* ≤ 0.001		
Rural	532 (28.14)	393 (37.71)
Urban	1377 (71.86)	737 (62.29)

Age at first intercourse		
*X* = 0.6218, *p* ≤ 0.05		
<16	589 (30.49)	347 (31.86)
16+	1320 (69.51)	783 (68.14)

Knowledge of fertile period		
*X* = 14.7421, *p* ≤ 0.001		
Yes	1710 (90.25)	1058 (94.23)
No	199 (9.75)	72 (5.77)

Registered with health insurance		
*X* = 3.3315, *p* > 0.05		
Yes	1543 (80.52)	941 (83.19)
No	366 (19.48)	189 (16.81)

Gravidae		
*X* = 18.8832, *p* ≤ 0.001		
1–3	760 (39)	516 (44.67)
4–5	590 (32.56)	29 (25.24)
6+	559 (28.44)	323 (30.1)

**Table 3:** Univariate and multivariate logistic analyses of predictors of contraceptives use among women who ever had induced abortion (15–49 years).

Variable category	Univariate OR (95% CI), *p* value	Multivariate aOR (95% CI), *p* value
Sociodemographic		
Age of respondents		
15–19	1	1
20–24	1.01 (0.62–1.63), 0.979	0.97 (0.60–1.57), 0.903
25–29	0.84 (0.52–1.35), 0.467	0.76 (0.46–1.25), 0.279
30–34	0.61 (0.38–0.98), 0.043	0.50 (0.30–0.85), 0.010
35–39	0.51 (0.31–0.83), 0.008	0.35 (0.20–0.62), 0.000
40–44	0.37 (0.22–0.616), 0.000	0.25 (0.14–0.45), 0.000
45–49	0.30 (0.18–0.48), 0.000	0.22 (0.12–0.41), 0.000
Age at first intercourse		
<16	1	
16 or above	0.94 (0.77–1.14), 0.519	
Union		
No	1	1
Yes	1.32 (1.09–1.59), 0.004	1.45 (1.20–1.75), 0.000
Religious affiliation		
Christianity	1	
Islam	1.02 (0.69–1.52), 0.906	
Traditional	1.734 (0.40–7.56), 0.463	
Others	1.17 (0.54–2.53), 0.693	
Region		
Central	1	1
Western	1.75 (1.12–2.75), 0.015	1.58 (1.00–2.49), 0.049
Ashanti	1.820 (1.21–2.74), 0.004	1.87 (1.25–2.80), 0.002
Northern	2.20 (1.19–4.08), 0.012	1.98 (1.05–3.72), 0.034
Brong ahafo	2.06 (1.28–3.31), 0.003	2.07 (1.31–3.26), 0.002
Upper West	1.06 (0.53–2.13), 0.876	0.92 (0.44–1.90), 0.819
Upper East	1.61 (0.80–3.27), 0.184	1.50 (0.72–3.13), 0.284
Greater accra	1.12 (0.72–1.73), 0.612	1.37 (0.87–2.15), 0.175
Volta	1.52 (0.93–2.48), 0.093	1.60 (0.97–2.62), 0.175
Eastern	2.27 (1.47–3.49), 0.000	2.32 (1.50–3.60), 0.000

Socioeconomic characteristics		
Educational level		
Primary	1	1
JHS	1.03 (0.80–1.54), 0.821	0.87 (0.66–1.14), 0.322
SHS or above	0.74 (0.55–0.99), 0.043	0.79 (0.56–1.11), 0.173
No education	1.11 (0.80–1.54), 0.542	1.04 (0.73–1.48), 0.831
Place of residence		
Urban	1	1
Rural	1.55 (1.26–1.90), 0.000	1.31 (1.04–1.65), 0.022
Registered with health insurance		
Yes	1	
No	0.84 (0.68–1.03), 0.086	
Abortion-related characteristics		
Gravidae	1	1
1–3	1	1
4–5	0.68 (0.55–0.84), 0.000	0.95 (0.75–1.23), 0.733
6 or more	0.92 (0.75–1.14), 0.454	1.74 (1.35–2.23), 0.000
Knowledge of the fertile period		
No	1	1
Yes	1.63 (1.08–2.46), 0.020	1.84 (1.30–2.60), 0.001

OR, odds ratio. aOR, adjusted odds ratio. CI, confidence interval. Goodness of fittest. F-adjusted test statistic = F (9,680) = 0.84. Prob > F = 0.5748.

## Data Availability

The datasets generated and/or analyzed during the current study are available in the Ghana demographic and health repository, http://dhsprogram.com/data/available-datasets.cfm.

## References

[1] Ganatra B., Gerdts C., Rossier C. (2017). Global, regional, and subregional classification of abortions by safety, 2010-14: estimates from a Bayesian hierarchical model. *The Lancet*.

[2] Shah I. H., Åhman E., Ortayli N. (2014). Access to safe abortion: progress and challenges since the 1994 international conference on population and development (ICPD). *Contraception*.

[3] Keogh S. C., Otupiri E., Chiu D. W. (2020). Estimating the incidence of abortion : a comparison of five approaches in ghana. *BMJ Global Health*.

[4] Rominski S. D., Lori J. R. (2014). Abortion care in Ghana: a critical review of the literature. *African Journal of Reproductive Health*.

[5] Morhee R. A. ., Morhee E. S. (2010). Overview of the law and availability of abortion services in Ghana. *Ghana Medical Journal*.

[6] Atakro C. A., Addo S. B., Aboagye J. S. (2019). Contributing factors to unsafe abortion practices among women of reproductive age at selected district hospitals in the Ashanti region of Ghana. *BMC Women’s Health*.

[7] Amalba A., Mogre V., Appiah M. N. A., Mumuni W. A. (2014). Awareness, use and associated factors of emergency contraceptive pills among women of reproductive age (15–49 years) in Tamale, Ghana. *BMC Women’s Health*.

[8] Apanga P. A., Adam M. A. (2015). Factors influencing the uptake of family planning services in the Talensi district, Ghana. *Pan African Medical Journal*.

[9] Geelhoed D. W., Nayembil D., Asare K., Van Leeuwen J. H. S., Van Roosmalen J. (2002). Contraception and induced abortion in rural Ghana. *Tropical Medicine and International Health*.

[10] Ahmed S., Li Q., Liu L., Tsui A. O. (2012). Maternal deaths averted by contraceptive use: an analysis of 172 countries. *The Lancet*.

[11] Agyemang J., Newton S., Nkrumah I., Tsoka-Gwegweni J. M., Cumber S. N. (2019). Contraceptive use and associated factors among sexually active female adolescents in Atwima Kwanwoma District, Ashanti region-Ghana. *Pan African Medical Journal*.

[12] Aviisah P. A., Dery S., Atsu B. K. (2018). Modern contraceptive use among women of reproductive age in Ghana: analysis of the 2003–2014 Ghana demographic and health surveys. *BMC Women’s Health*.

[13] Grindlay K., Dako-gyeke P., Ngo T. D. (2018). Contraceptive use and unintended pregnancy among young women and men in Accra, Ghana. *PLoS One*.

[14] Kebede A., Abaya S. G., Merdassa E., Bekuma T. T. (2019). Factors affecting demand for modern contraceptives among currently married reproductive age women in rural Kebeles of Nunu Kumba district, Oromia, Ethiopia. *Contraception and Reproductive Medicine*.

[15] Nonvignon J., Novignon J. (2014). Trend and determinants of contraceptive use among women of reproductive age in Ghana. *Etude de La Population Africaine*.

[16] Nyarko S. H. (2020). Spatial variations and socio-economic determinants of modern contraceptive use in Ghana : a Bayesian multilevel analysis. *PLoS One*.

[17] Lamina M. A. (2015). Prevalence of abortion and contraceptive practice among women seeking repeat induced abortion in Western Nigeria. *Journal of Pregnancy*.

[18] Makenzius M., Faxelid E., Gemzell-Danielsson K., Odero T. M. A., Klingberg-Allvin M., Oguttu M. (2018). Contraceptive uptake in post abortion care—secondary outcomes from a randomised controlled trial, Kisumu, Kenya. *PLoS One*.

[19] Mekuria A., Gutema H., Wondiye H., Abera M. (2019). Postabortion contraceptive use in Bahir Dar, Ethiopia: a cross sectional study. *Contraception and Reproductive Medicine*.

[20] Opoku B. (2012). Contraceptive preferences of post-abortion patients in Ghana. *Journal of Women’s Health Care*.

[21] Ghana Statistical Service (GSS) (2017). *Ghana Health Service (GHS), and ICF Macro. Accra: Ghana Maternal and Health Survey*.

[22] Archer K. J., Lemeshow S. (2006). Goodness-of-fit test for a logistic regression model fitted using survey sample data. *The Stata Journal: Promoting Communications on Statistics and Stata*.

[23] Ceylan A., Ertem M., Saka G., Akdeniz N. (2009). Post abortion family planning counseling as a tool to increase contraception use. *BMC Public Health*.

[24] Abebe A. M., Wudu Kassaw M., Zemariam A. B., Estifanos Shewangashaw N. (2019). Coverage, opportunity, and challenges of expanded program on immunization among 12–23-month-old children in woldia town, northeast Ethiopia, 2018. *BioMed Research International*.

[25] Maxwell L., Voetagbe G., Paul M., Mark A. (2015). Does the type of abortion provider influence contraceptive uptake after abortion? An analysis of longitudinal data from 64 health facilities in Ghana. *BMC Public Health*.

[26] Chae S., Desai S., Crowell M., Sedgh G., Singh S. (2017). Characteristics of women obtaining induced abortions in selected low- and middle-income countries. *PLoS One*.

[27] Håkansson M., Oguttu M., Gemzell-Danielsson K., Makenzius M. (2018). Human rights versus societal norms: a mixed methods study among healthcare providers on social stigma related to adolescent abortion and contraceptive use in Kisumu, Kenya. *BMJ Global Health*.

[28] Asrat M., Bekele D., Rominski S. D. (2018). Post-abortion contraceptive acceptance and choice determinants among women receiving abortion care at saint Paul’s hospital, Addis Ababa, Ethiopia. *Ethiopian Journal of Reproductive Health (EJRH)*.

[29] EK E., Abera A. (2016). Safe abortion care, utilization of post abortion contraception and associated factors, jimma Ethiopia. *Journal of Women’s Health Care*.

[30] Kokeb L. (2015). Utilization of post abortion contraceptive and associated factors among women who came for abortion service: a hospital based cross sectional study. *Journal of Family Medicine and Disease Prevention*.

[31] Postlethwaite D., Lee J., Merchant M., Alabaster A., Raine-Bennett T. (2018). Contraception after abortion and risk of repeated unintended pregnancy among health plan members. *The Permanente Journal*.

[32] Gonie A., Wudneh A., Nigatu D., Dendir Z. (2018). Determinants of family planning use among married women in bale eco-region, Southeast Ethiopia: a community based study. *BMC Women’s Health*.

[33] Buor D. (2004). Determinants of utilisation of health services by women in rural and urban areas in Ghana. *GeoJournal*.

[34] Johnston H. B., Ved R., Lyall N., Agarwal K. (2003). Where do rural women obtain postabortion care? The case of Uttar Pradesh, India. *International Family Planning Perspectives*.

[35] Adanu R. M., Seffah J., Anarfi J. K., Lince N., Blanchard K. (2012). Sexual and reproductive health in Accra, Ghana. *Ghana Medical Journal*.

[36] Amazigo U., Silva N., Kaufman J. (1997). Nigeria sexual activity and contraceptive knowledge and use among in-school adolescents in Nigeria. *International Family Planning Perspectives*.

[37] Samuel M., Fetters T., Desta D. (2016). Strengthening postabortion family planning services in Ethiopia: expanding contraceptive choice and improving access to long-acting reversible contraception. *Global Health: Science and Practice*.

